# Focal Choroidal Elevations: Localized Pigment Epithelial Contour Alterations due to Isolated Choroidal Vessels

**DOI:** 10.1155/2019/4962363

**Published:** 2019-10-07

**Authors:** Rachel L. Chu, Nicole A. Pannullo, Eric J. Sigler

**Affiliations:** ^1^Stony Brook University School of Medicine, 101 Nicolls Road, Health Sciences Center, Level 4, Stony Brook, NY 11794, USA; ^2^School of Chemistry and Materials Science, Rochester Institute of Technology, 1 Lomb Memorial Drive, Rochester, NY 14623, USA; ^3^Division of Retina and Vitreous, Ophthalmic Consultants of Long Island, 2000 North Village Avenue, Suite 402, Rockville Center, NY 11570, USA

## Abstract

**Purpose:**

The objective of this case series was to describe the clinical and imaging features of focal choroidal elevations (FCE), which are chorioretinal contour changes induced by individual choroidal vessels within an overall thin-appearing choroid.

**Methods:**

A total of 787 enhanced depth imaging (EDI) spectral domain optical coherence tomography (SD-OCT) patient images were initially screened for the presence of FCE. Prospective imaging analysis of 38 patients with FCE was done. Mean central macular choroidal thickness (CMCT), FCE location, FCE vessel lumen diameter, patient demographics, cycloplegic autorefraction, ophthalmoscopic findings, and presence of choroidal neovascularization (CNV) in the fellow eye were recorded.

**Results:**

FCE were observed in 25 patients with age-related macular degeneration (ARMD), in 5 patients with high myopia, and in 8 patients with age-related choroidal atrophy (ARCA). Mean patient age was 80 ± 9.4 years. Mean CMCT was 86 ± 40 *μ*m. Mean lumen diameter of the vessels inducing FCE was 131 ± 33 *μ*m.

**Conclusions:**

FCE are relatively frequently encountered morphologic features of elderly patients with ARMD, high myopia, and ARCA, and have a distinct clinical and imaging morphology which differs from classically described chorioretinal folds. The lesions may commonly be mistaken for pigment epithelial detachments on ophthalmoscopy, may be associated with CNV in fellow eyes, and have a characteristic SD-OCT appearance.

## 1. Introduction

The choroid, which is the middle vascular layer of the eye, is a lobular network of pigmented capillaries, is the efferent vascular extension of the short posterior ciliary arteries, and supplies greater than 90% of the oxygen consumption to the posterior ocular segment [1]. Histologic and imaging studies have indicated three major choroidal vascular layers: an outer large vessel layer (Haller's layer), a middle intermediate-sized vascular layer (Sattler's layer) with numerous ganglion cells [2], and an inner choriocapillaris, which terminates adjacent to Bruch's membrane and demonstrates fenestrated endothelium [3–5]. With recent advances in optical coherence tomography, particularly spectral domain optical coherence tomography (SD-OCT) and enhanced depth imaging (EDI) [6], the entire cross-sectional extent of the choroid is visible *in vivo*. This has led to many advances in the clinical ability to evaluate choroidal morphology in posterior segment disease.

Age-related choroidal atrophy (ARCA) is a recently described [7] process by which the choroid becomes progressively thinner with advancing age and may be associated with features of age-related macular degeneration (ARMD), such as drusen and subretinal drusenoid deposits [8, 9]. The clinical and anatomic associations with ARCA may be incompletely described, in part due to the numerous factors, such as axial length, medications [10], systemic blood flow alterations, diabetes [11], underlying chorioretinal pathology, and potentially unknown factors, which may affect choroidal thickness. Patients with high myopia, ARCA, and retinal pigment epithelial cell loss [12, 13] appear to have a particularly thin choroid. In addition, the authors have recently observed a relatively thin choroid in patients over age 65 with early ARMD and in patients with a history of cigarette smoke exposure [14, 15].

Chorioretinal contour changes within the macula are a well-known cause of visual impairment and include retinal pigment epithelial detachments (PED), chorioretinal folds from idiopathic or secondary etiologies [16–18], and pathologic myopia [19]. Choroidal excavations and intrachoroidal cavitations are additional morphologic features of the choroid visible with SD-OCT which may be observed in association with intermediate and high myopia [20–22]. The authors have recently observed several patients who have a focal retinal pigment epithelial elevation which corresponds to large-sized or medium-sized choroidal vessels in the context of an overall thin-appearing choroid on EDI SD-OCT. The lesions may have a “pseudo-PED” appearance on ophthalmoscopy and appear to have similar features to PED and chorioretinal folds. The purpose of the present study was to describe the morphology and clinical features of these focal choroidal elevations (FCEs).

## 2. Materials and Methods

This prospective, consecutive, and observational case series conformed to the tenets set forth in the Declaration of Helsinki and was performed in accordance with the Health Insurance Portability and Accountability Act of 1996. The present series represents a subset of patients evaluated during an ongoing IRB-approved clinical study, Choroidal Thickness in Age-Related Eye Disease. All patients were seen and evaluated by vitreoretinal specialists at the Charles Retina Institute in Memphis, Tennessee, over a two-month consecutive period in 2013. Patients were evaluated with mydriatic ophthalmoscopy, fundus photography (VisuCam, Carl Zeiss Meditec, Dublin, California), fundus autofluorescence, EDI SD-OCT, and fluorescein angiography (Spectralis, Heidelberg Engineering, Heidelberg, Germany).

Patient imaging was evaluated by a single observer, the author Eric J. Sigler (EJS). EDI imaging consisted of 12-line radial raster scanning; 20 scans averaged/raster were obtained in all patients. SD-OCT images were initially screened for the presence of FCEs. Patients with FCEs were further analyzed on the day of their regularly scheduled office visit. Cycloplegic autorefraction on the day of the exam was entered into the preimaging patient data screen. For mean central macular choroidal thickness (CMCT) measurements, manual axial values were obtained at the foveola and at 6 additional 500 *μ*m intervals in the central 3000 *μ*m of the macula for each raster by a single observer, the author EJS (total 84 points/eye). The device caliper tool was used to extend a line from the inner scleral border to Bruch's membrane or outer border of the retinal pigment epithelium (RPE). The values were then averaged for the mean CMCT value. Axial FCE lumen diameters were included in the overall average CMCT.

Scans with poor resolution due to media opacity were initially excluded (8 patients). Data including CMCT, FCE location (quadrant of lesion), patient demographics, cycloplegic autorefraction (spherical equivalent in diopters), ophthalmoscopic findings, and presence of choroidal neovascularization (CNV) in the fellow eye were recorded. To be considered an FCE, the lesion had to involve a distinct chorioretinal contour elevation on both sides of the choroidal vessel without overlying drusen or additional pathology as viewed on a cross-sectional, grey-scale, B-scan image. All FCEs were visible on at least two adjacent raster scans. The FCE vessel lumen diameter was calculated by measuring the axial lumen diameter with the device caliper tool. The author EJS instructed Amsler grid testing to all patients with FCEs on the day of the exam.

## 3. Results

**Figure 1:**
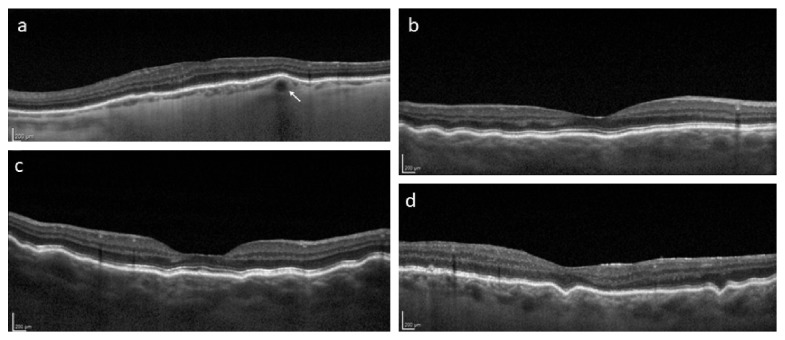
Spectral-domain optical coherence tomography findings in focal choroidal elevations and chorioretinal folds. (a) Left eye of a 79-year-old male with age-related choroidal atrophy, but no additional eye pathology demonstrating a focal chorioretinal contour change overlying a large choroidal vessel (arrow) or focal choroidal elevation; mean central macular choroidal thickness = 48 *μ*m. (b) Right eye of a patient with idiopathic uveal effusion syndrome demonstrating chorioretinal folds overlying a relatively thick choroid that do not correspond to specific choroidal vessels. (c) Right eye of a patient with chorioretinal folds due to external globe compression from a lacrimal adenocarcinoma. Chorioretinal folds do not correspond to specific choroidal vessels as in (d) the right eye of patient with an encircling scleral buckle and chorioretinal folds.

**Figure 2:**
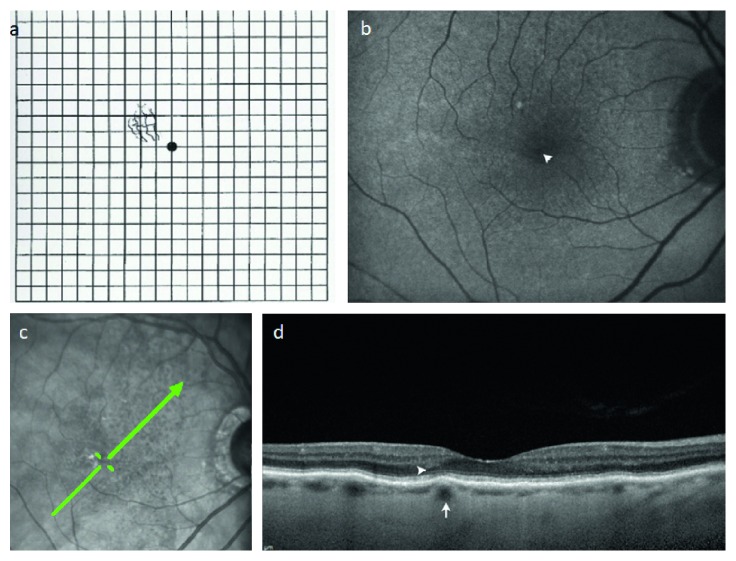
Metamorphopsia and focal choroidal elevations in a patient with atrophic age-related macular degeneration. (a) Amsler grid demonstrating metamorphopsia drawn by this 86-year-old female patient. (b) Right eye fundus autofluorescence with no evidence of geographic atrophy but showing a faint pinpoint area of relative hypoautofluorescence (arrowhead) corresponding to focal choroidal elevation in (c) scanning laser ophthalmoscopic image with green arrow showing raster position for SD-OCT in (d) focal choroidal elevation (arrow) without overlying drusen or atrophy corresponds to the location of metamorphopsia; there is subtle disruption of the ellipsoid portion of photoreceptor outer segments (IS/OS line) overlying this region (arrowhead).

Seven hundred eighty-seven patient images were evaluated over the two-month study period. 38 eyes of 38 patients (4.8%) demonstrated FCEs and were included for further analysis. Overall mean age ± standard deviation was 80 ± 9.4 years and consisted of 26 females and 12 males. Overall mean BCVA was 0.29 ± 0.20 logMAR units. 34/38 patients were pseudophakic. Mean CMCT was 86 ± 40 *μ*m (range = 24–198 *μ*m). All FCEs had a distinct appearance on EDI SD-OCT, involved a specific choroidal vessel beneath overlying chorioretinal contour changes, and appeared different from true chorioretinal folds, as demonstrated in Figure 1. The lesions appeared as PED in 36/38 patients on ophthalmoscopy. Overall mean lumen diameter of the vessels inducing FCEs was 131 ± 33 *μ*m (range = 78–239 *μ*m). Underlying diagnoses included ARMD (*n* = 25), pathologic myopia (*n* = 5), and idiopathic (*n* = 8). FCEs were located at the following locations: subfoveal (*n* = 19), superotemporal macula (*n* = 7), inferotemporal macula (*n* = 3), within the temporal horizontal raphe (*n* = 5), superonasal macula (*n* = 3), or inferonasal macula (*n* = 1). 27/38 (71%) patients had a history of choroidal neovascularization in their fellow eye. FCEs were single in 21/38 eyes, multiple in 17/38 eyes, and bilateral in 15/38. FCEs were documented as PED in 36/38 lesions. Fluorescein angiography demonstrated that FCEs filled earlier than adjacent choroid and were completely filled during the retinal arterial phase, indicating arterial origin. Metamorphopsia in the absence of additional pathology was demonstrated in 17/38 (55%), all of which were subfoveal, as demonstrated in Figure 2. Overall, 17/19 (89%) of subfoveal FCEs were associated with metamorphopsia.

### 3.1. Age-Related Macular Degeneration

**Figure 3:**
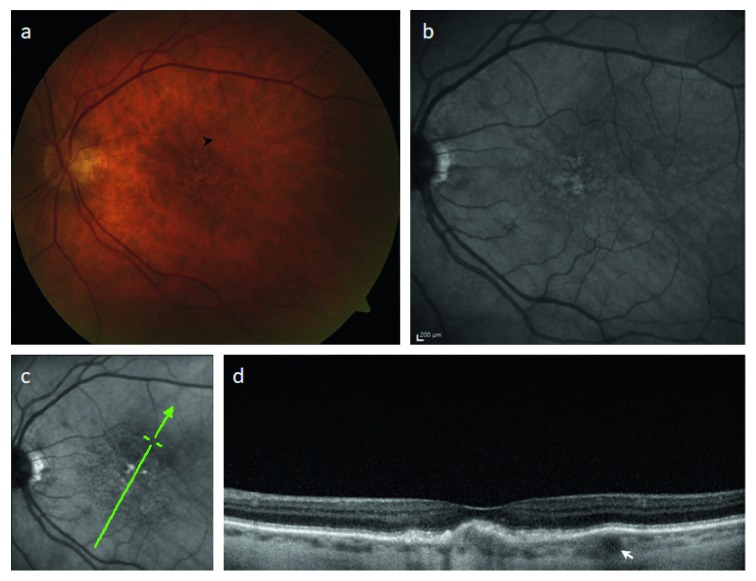
Imaging features of a small focal choroidal elevation in a patient with early atrophic age-related macular degeneration. (a) Color fundus photograph demonstrating drusen and the position of a subtle pigment epithelial elevation (arrowhead) observed on ophthalmoscopy. (b) Fundus autofluorescence reveals no specific abnormality in the region. (c) Scanning laser ophthalmoscopic image; green arrow corresponding to SD-OCT raster in (d). There is a subfoveal drusenoid pigment epithelial detachment and a focal choroidal elevation (FCE) corresponding to the region observed in (a); mean macular choroidal thickness = 105 *μ*m; FCE lumen diameter = 126 *μ*m.

**Figure 4:**
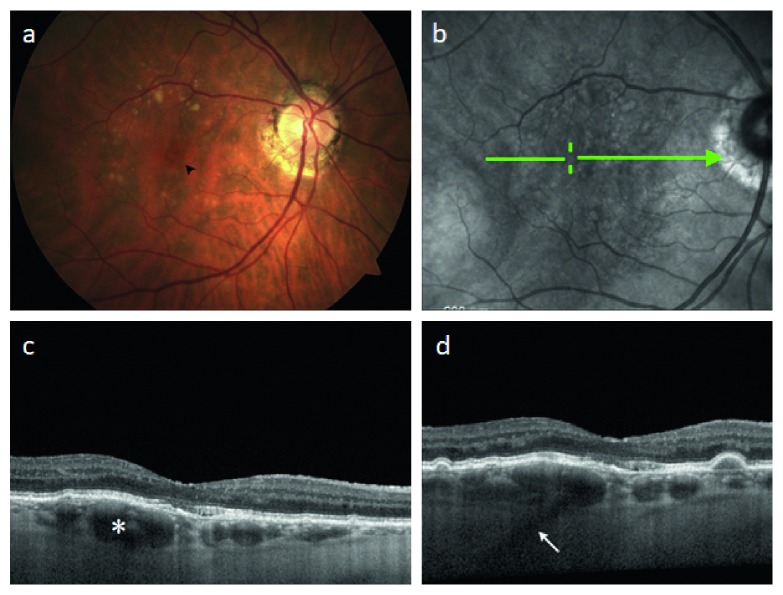
Imaging characteristic of a large focal choroidal elevation simulating a pigment epithelial detachment in atrophic age-related macular degeneration. (a) Color fundus photograph demonstrating drusen and an orange-red foveal lesion which appeared elevated on ophthalmoscopy. (b) Scanning laser ophthalmoscopic image with green arrow corresponding to raster position for SD-OCT in (c) revealing a large focal choroidal elevation (FCE); lumen = 239 *μ*m which corresponds to the lesion in (a). (d) Adjacent raster scan reveals evidence of a posterior ciliary vessel continuous with the FCE (arrow).

Twenty-five patients with FCEs had an underlying diagnosis of atrophic ARMD in the studied eye. Mean age in this subgroup was 82 ± 7.3 years. Mean spherical equivalent was +0.62 ± 0.51 diopters. Mean CMCT was 93 ± 37 *μ*m, and mean lumen diameter was 129 ± 32 *μ*m. Mean BCVA was 0.30 ± 0.18. 21 patients had a history of choroidal neovascularization in their fellow eye. FCE location was as follows: subfoveal (*n* = 14), superotemporal (*n* = 4), temporal horizontal raphe (*n* = 2), inferotemporal (*n* = 1), superonasal (*n* = 3), or inferonasal (*n* = 1). One subfoveal lesion was asymptomatic. Fundus autofluorescence demonstrated the absence of RPE atrophy overlying FCEs. Examples of patient images with FCEs and typical features of atrophic ARMD are presented in Figures 3 and 4.

### 3.2. High Myopia

**Figure 5:**
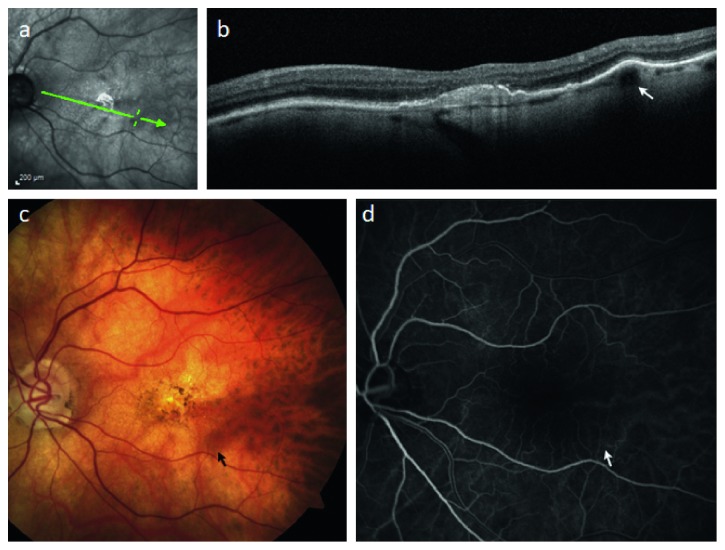
Imaging characteristics of a focal choroidal elevation in a patient with pathologic myopia. (a) Scanning laser ophthalmoscopic image; green arrow corresponds to SD-OCT in (b) demonstrating a focal choroidal elevation in an overall thin choroid (mean macular choroidal thickness = 24 *μ*m). (c) Color fundus photograph reveals dusky red-colored lesion that resembles a pigment epithelial detachment on ophthalmoscopy corresponding to the FCEs in (b). (d) Laminar phase fluorescein angiogram reveals a complete fill of this choroidal vessel which appeared to originate from the location of the FCEs, indicating its probable direct continuation from a short posterior ciliary artery.

Five patients with FCEs had an underlying diagnosis of high myopia. Mean age in this subgroup was 70 ± 15 years and was significantly younger than both the ARMD and idiopathic subgroups via one-way ANOVA (*p*-value = 0.023). The mean spherical equivalent in this group (cycloplegic autorefraction in phakic patients or prior to cataract extraction in pseudophakic patients) was −12 ± 3.2 diopters. All patients in this group had ophthalmoscopic features of pathologic myopia. Mean BCVA was 0.38 ± 0.31 logMAR units. Mean CMCT was 51 ± 40 *μ*m, and mean FCE lumen diameter was 115 ± 34 *μ*m. One patient had a history of choroidal neovascularization in their fellow eye. FCE location was as follows: subfoveal (*n* = 2), superotemporal (*n* = 1), inferotemporal (*n* = 1), or temporal horizontal raphe (*n* = 1). Both subfoveal lesions were associated with metamorphopsia on Amsler grid testing. Imaging features of FCEs in pathologic myopia are demonstrated in Figure 5.

### 3.3. Age-Related Choroidal Atrophy

Eight patients had no specific underlying chorioretinal pathology and were therefore considered idiopathic. Mean age was 81 ± 8.0 years, and mean CMCT was 85 ± 43 *μ*m; therefore, this subgroup represented ARCA. Mean FCE lumen diameter was 143 ± 38 *μ*m. Mean spherical equivalent was −0.54 ± 0.65 diopters. BCVA was 0.20 ± 0.14 logMAR units. 2/8 patients had choroidal neovascularization in their fellow eye, one with peripapillary CNV without polypoidal features and one with subfoveal CNV without drusen, RPE atrophy, or any indication of an additional inciting lesion. FCE location was as follows: subfoveal (*n* = 3), superotemporal (*n* = 2), inferotemporal (*n* = 1), or temporal horizontal raphe (*n* = 2). One subfoveal FCE was asymptomatic.

## 4. Discussion

The observations in the present series indicate that chorioretinal contour changes overlying presumably medium-sized choroidal vessels are relatively common SD-OCT features in patients with high myopia and ARMD and may be seen in the context of ARCA without additional obvious eye disease. While we observed FCEs in 4.8% of patients in the present series, this likely overestimates the true incidence of the lesion due to the evaluation of patients with underlying chorioretinal disease and the large number of patients with ARMD presenting to a vitreoretinal specialist. Nevertheless, the finding was a consistent feature observed in patients with the above diagnoses and, to the author's knowledge, has not been previously reported.

Choroidal varices, chorioretinal folds, and varices of vortex vein ampullae are additional previously described posterior segment findings with some similar features to FCEs [17, 18, 23–25]. In contrast to chorioretinal folds (Figure 1), FCEs appear to be the result of individual, intermediate-sized choroidal vessels located immediately adjacent to the underlying RPE. Chorioretinal folds appear to involve choroidal and retinal contour changes, which do not correspond to specific choroidal vessels and have a distinct appearance on SD-OCT. Chorioretinal folds have been described in ARMD [17, 18, 23, 26]; however, we did not observe true chorioretinal folds, as occurring in hypotony, external globe compression, or idiopathic uveal effusion in patients with FCEs. There is a paucity of published data examining chorioretinal fold morphology using SD-OCT. A previous report using time-domain OCT [23] identified chorioretinal contour changes in 8 patients; however, close inspection reveals that at least one of these [23] corresponds to specific medium-sized choroidal vessels (FCE) in an adult with ARMD. We therefore suggest that at least a subset of previously reported “chorioretinal folds” actually correspond to individual choroidal vessels in the context of an overall atrophic choroid when seen in association with ARMD. The previous underrecognition of FCEs likely results from decreased resolution of previously available imaging devices, such as time-domain OCT.

Common morphologic features present in patients with FCEs included a markedly thin choroid, typical feature of ARMD or pathologic myopia and the presence of CNV in fellow eyes. FCEs have a similar appearance to choroidal varices [24, 25], and the authors have recently observed a symptomatic subfoveal choroidal varix with an FCE appearance. While FCEs may be associated with additional pathologic features such as drusen, RPE atrophy, and choroidal neovascularization, the present series identified numerous patients with metamorphopsia and no overlying pathology in the context of a subfoveal FCE, indicating that the lesions may induce visual symptoms in the absence of specific additional structural pathology. Potential mechanisms by which medium-sized choroidal vessels may produce chorioretinal contour changes include primary anatomic variants, dilation of choroidal vessels, or overall atrophy of choriocapillaris to a greater extent than larger choroidal vessels. In the present series, the mean overall CMCT was thinner than mean FCE lumen diameter and, in fact, this was the case in 36 of 38 patients analyzed. In addition, we found no evidence of choroidal vessels with a particularly large lumen, no evidence of choroidal thickening, and no evidence of systemic disease associated with vascular dilation in patients with FCEs. Fluorescein angiography demonstrated early filling of FCE vessels, and both angiography and EDI revealed the pseudo-PED appearance present overlying the origin of the choroidal vessel, frequently continuous with a posterior ciliary vessel. The authors therefore hypothesize that progressive choroidal atrophy may lead to preferential loss of the choriocapillaris and may induce FCEs as the RPE and the retina become draped over the more rigid, remaining arterial outer choroidal vessels. Visual symptoms may result secondary to retinal contour changes overlying these regions and may also be related to atrophy of the underlying choriocapillaris, which manifests as metamorphopsia in subfoveal FCEs.

The present study is limited by its single-center and cross-sectional design, predominantly Caucasian study population, and lack of formal statistical analysis due to a relatively small sample size. In addition, we did not routinely perform indocyanine green angiography, which may have revealed additional angiographic details of FCEs.

## 5. Conclusions

We conclude that FCEs are relatively frequently encountered morphologic features of elderly patients with ARMD, high myopia, and ARCA and have a distinct clinical and imaging morphology which differs from classically described chorioretinal folds. The lesions may commonly be mistaken for pigment epithelial detachments on ophthalmoscopy, may be associated with CNV in fellow eyes, and have a characteristic SD-OCT appearance.

## Data Availability

The data used to support the findings of this study are included within the article.
